# Quality of life in patients with retinitis pigmentosa submitted to intravitreal use of bone marrow-derived stem cells (Reticell -clinical trial)

**DOI:** 10.1186/s13287-015-0020-6

**Published:** 2015-03-14

**Authors:** Rubens C Siqueira, Andre Messias, Katharina Messias, Rafael S Arcieri, Milton A Ruiz, Neiglene F Souza, Lia C Martins, Rodrigo Jorge

**Affiliations:** Department of Ophthalmology, Otorhinolaryngology and Head and Neck Surgery, School of Medicine of Ribeirão Preto, University of São Paulo, Ribeirão Preto, Brazil; Rubens Siqueira Research Center, Sao Jose do Rio Preto, SP Brazil; Discipline of Biology, Paulista University (UNIP), Sao Jose do Rio Preto, SP Brazil; Department of Bone Marrow Transplantation, Beneficencia Portuguesa Hospital, Sao Jose do Rio Preto, SP Brazil; Hospital das Clinicas da Faculdade de Medicina de Ribeirao Preto, Avenida Bandeirantes, 3900, Ribeirao Preto, SP 14049-900 Brazil

## Abstract

**Introduction:**

Retinitis pigmentosa (RP) is a severe neurodegenerative disease of the retina that can lead to blindness. Even without treatment, a clinical study with the use of stem cells is currently underway and the results are being evaluated. In the present report we assess the vision-related quality of life in patients with RP submitted to intravitreal use of bone marrow-derived stem cells.

**Method:**

The study included 20 patients with RP submitted to intravitreal use of bone marrow-derived stem cells. We evaluate the vision-related quality of life (VRQOL) of patients using the National Eye Institute Visual Function Questionnaire-25 (NEI VFQ-25). Patients were scheduled to answer the questionnaire before treatment and 3 and 12 months after treatment.

**Results:**

All patients completed the survey as scheduled.

There was a statistically significant improvement (P <0.05) in the quality of life of patients 3 months after treatment, whereas by the 12th month there was no statistically significant difference from baseline.

**Conclusions:**

Cell therapy with intravitreal use of bone marrow-derived stem cells can improve the quality of life of patients with RP, although the improvement is lost with time. A larger number of cases will be necessary to evaluate the repercussions of this therapy on the quality of life of these patients.

**Trial registration:**

Clinicaltrials.gov: NCT01560715. Registered March 19, 2012.

## Introduction

Retinitis pigmentosa (RP) is a severe neurodegenerative disease of the retina initially characterised by night blindness, with progression to tunnel vision and eventual loss of central vision and total blindness. Targeted therapies for RP are complicated by the identification of more than 40 genes linked to the dominant and recessive forms of this disease [1,2].

A few new approaches to RP treatment have been recently investigated, including nutritional supplementation, light reduction and gene therapy; valproic acid and vitamin A supplementation which is the most promising, but its benefits are modest and side effects are problematic. Therefore, there is currently no significant treatment or cure for RP [3-6].

RP is emotionally devastating to the patients due to their anticipated vision loss, and the subsequent experience of gradual vision loss causes severe distress. These factors could make RP patients more vulnerable to depression, but few studies have addressed the issues of depression and the vision-related quality of life in this population [6].

Three clinical trials are being conducted in Brazil by our research group on the use of autologous, bone marrow-derived, hematopoietic stem cell transplantation (auto-BMHSCT) for the treatment of RP, dry age-related macular degeneration and ischaemic retinopathy (including diabetic retinopathy with macular ischaemia) [7]. These studies are registered with ClinicalTrials.gov, numbers NCT01068561, NCT01518127 and NCT01518842, respectively. We published the results of phase I of the clinical trial using an intravitreal injection of BM-derived hematopoietic stem cells (CD34 +) for retinal dystrophy (including RP) [8]. The second phase of this study (NCT01560715) has already begun [9].

In the present report, we assess the quality of life status of patients with RP submitted to intravitreal use of BM-derived stem cells (Retinacell- clinical trial – phase 2) using the National Eye Institute Visual Function Questionnaire (NEI-VFQ).

## Methods

The study protocol adhered to the tenets of the Declaration of Helsinki and was approved by the National Commission on Ethics in Research (CONEP) connected to the National Health Council (CNS) of Brazil and registered in clinicaltrial.gov: NCT01560715.

The study was conducted in a single center (Hospital das Clinicas, Medical School Ribeirao Preto- Sao Paulo- Brazil). All participants gave written informed consent. Patients were evaluated at the Retina and Vitreous Section of the Department of Ophthalmology, Otorhinolaryngology and Head and Neck Surgery, School of Medicine of Ribeirão Preto, between April 2012 and July 2013.

Throughout the study, a single certified examiner performed Early Treatment Diabetic Retinopathy Study best-corrected visual acuity (BCVA) measurement before any other study procedure. Ophthalmic evaluation was performed by a single retinal specialist (A.M.), and stereoscopic fundus photography, fluorescein and indocyanine green (ICG) angiography, microperimetry and optical coherence tomography (OCT) were performed by a single certified ophthalmic technician.

### Patient eligibility

Patients were included if they had: 1) a diagnosis of hereditary retinal dystrophy classified clinically as RP or cone–rod dystrophy, and 2) Early Treatment Diabetic Retinopathy Study BCVA of 20/200 (or worse) or visual field less than 20 degrees, considered legally blind. Exclusion criteria were: 1) previous ocular surgery other than cataract extraction; 2) presence of cataract or other media opacity that would prohibit high-quality ocular imaging or that would affect electroretinography (ERG) or visual field evaluation; 3) presence of other ophthalmic disease, such as glaucoma or uveitis; 4) history of blood disorders, such as leukemia; 5) known allergy to fluorescein or ICG angiography; or 6) known coagulation abnormalities or current use of anticoagulative medication other than aspirin. If both eyes were eligible for treatment, the eye with worse visual acuity was included in the study.

### Preparation of autologous bone marrow–derived stem cells

Aspiration of autologous bone marrow cells was performed under local anesthesia. Bone marrow (10 ml) was harvested from the posterior iliac crest and mononuclear cells were separated by Ficoll–Hypaque gradient centrifugation and suspended in buffered saline containing 5% human albumin at a concentration of 1 × 10^7^ cells/ml. The final product demonstrated absence of microbial contamination.

The final 0.1 ml of cell suspension used for the intravitreal injection contained 0.92 × 10^4^ to 2.91 × 10^4^ (mean: 1.68 × 10^4^) bone marrow–derived hematopoietic stem cells (CD34+).

### Intravitreal injection technique

All patients received an intravitreal injection using topical proparacaine drops under sterile conditions (eyelid speculum and povidone–iodine). Autologous (freshly isolated) bone marrow–derived mononuclear cells were injected into the vitreous cavity using a 27-gauge needle inserted through the inferotemporal pars plana 3.0 mm to 3.5 mm posterior to the limbus.

After the injection, central retinal artery perfusion was confirmed by indirect ophthalmoscopy. Patients were instructed to instill one drop of 0.3% ciprofloxacin into the injected eye four times daily for one week after the procedure.

The sham stem cell injection control procedure involved anaesthetizing the contralateral eye in a manner identical to that used for stem cell intravitreal injection. The tip of a needleless syringe was then pressed against the conjunctiva and the plunger of the needleless syringe depressed.

### Visual functioning questionnaire

The NEI-VFQ was used to evaluate the patients’ subjective visual function [10,11]. The NEI-VFQ-25 gives an overall score, as well as 12 subscale scores: general health, general vision, near vision, distance vision, driving, peripheral vision, colour vision, ocular pain, vision-associated role limitations, dependency, social functioning, and mental health. The questionnaire focuses on vision difficulties in everyday life, as well as vision-relevant psychosocial domains, such as mental health, social function and role difficulty. The patients filled out the package including the questionnaire for demographic information and NEI-VFQ, their responses were checked at the scheduled visit by a psychologist and if any were missing, the patients were asked to fill them out.

The following data were collected: sex, age, education, profession, and origin. All patients were instructed about the objectives and methodology and gave written informed consent to participate.

The questionnaire comprises five-point scale ratings that were transformed into percentages (0% to 100%). Patients were scheduled to answer the questionnaire before treatment (baseline) and three and twelve months after treatment.

After consultation with the Biostatistics Service, it was decided to obtain the outcome by descriptive statistical analysis. A *P* value of 0.05 was considered to be significant in all analyses.

## Results

All patients completed the survey as scheduled. We used the Student’s t-test for statistical calculation.

There was a statistically significant improvement (*P* <0.05) in the quality of life of patients three months after treatment, whereas by the twelfth month there was no statistically significant difference from baseline.

**Figure 1:**
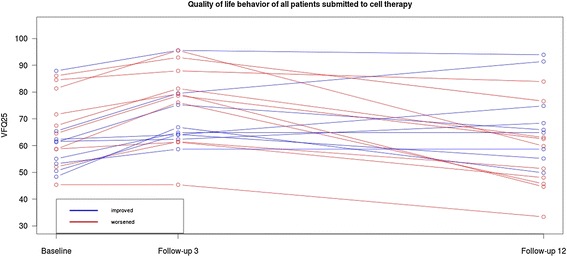
**Quality of life behaviour of all patients submitted to cell therapy.**

**Figure 2:**
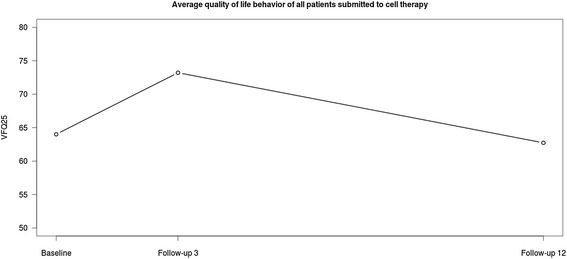
**Average quality of life behaviour of all patients submitted to cell therapy.**

Figure 1 illustrates the behaviour of quality of life of the patients based on the replies to the survey three to twelve months after treatment, and Figure 2 shows a line representing the average of all patients, more clearly revealing the significant improvement by the third month after treatment and the lack of a statistically significant improvement by the twelfth month after treatment.

## Discussion

In this article, we analyse the quality of life of patients with RP submitted to cell therapy (Intravitreal Autologous Bone-Marrow Stem Cells) using the NEI-VFQ-25. This study is part of a multiple investigation in which we have previously evaluated the visual field, visual acuity, fluorescein angiography, OCT and microperimetry in these same patients [12].

In a previous part of this study, we also observed an improvement in microperimetry in these 20 patients in the third and twelfth month, possibly explaining the improvement in quality of life that we observed in the present analysis, which coincided with the findings of microperimetry. However, in another study we did not observe a decrease in retinal sensitivity in the twelfth month as was observed in the present analysis of quality of life [13,14]. Therefore, we believe that there is also a significant psychological component in these patients, because RP is a progressive degenerative disease that leads to vision loss with no effective treatment and the high expectations of these patients with respect to this new kind of therapy may have influenced these results.

The correlation between quality of life and the sensitivity of the retina in patients with RP has been reported in other studies [13,14].

Seo *et al*. [15] reported that functional visual score, functional field score, and functional acuity score of the American Medical Association guidelines were equally correlated to those of the self-reported visual function questionnaire (NEI VFQ-25) in RP patients. They suggested that visual quality cannot be explained only by visual acuity or visual fields in RP patients. Similarly, macular sensitivity is also an important indicator of quality of life in RP patients.

Sugawara *et al*. [16] demonstrated that vision-related quality of life measured with the NEI VFQ-25 was significantly correlated with macular sensitivity in RP patients whose visual acuity was relatively good. These results indicate that macular sensitivity determined by microperimetry (MP1) is a good predictor of quality of life in RP patients with relatively good vision [16].

Park *et al*. [17] experimentally evaluated the behaviour of bone marrow-derived stem cells when injected intravitreally with respect to survival and clinical effect. They determined that the intravitreally injected CD34 cells were present in the retinal vasculature in the fourth month after the injection and presented a clinical effect observed in the electroretinogram until the eighth month. These findings also concur with the clinical response that we observed in our study and may explain the fall in quality of life at the end of the first year. We believe that, due to the temporary effect of this technique in these cases of retinal degeneration, a new dose of intravitreal injection of stem cells is required for the maintenance of a clinical effect.

## Conclusions

The intravitreal use of bone marrow-derived stem cells for the treatment of RP can improve the quality of life of these patients at the third month visit but it deteriorates afterwards.

This improvement may be related to improved microperimetry and also to patient expectations. An analysis after the second intravitreal injection of stem cells, taken after the first year of treatment, may provide a better definition of the influence of improvement in retinal sensitivity (measured by microperimetry) and the psychological component (patient expectations) on the results of the quality of life test.

## Abbreviations

auto-BMHSCT: autologous, BM-derived, hematopoietic stem cell transplantation
BCVA: best-corrected visual acuity
ERG: electroretinography
ICG: indocyanine green
NEI-VFQ: National Eye Institute Visual Function Questionnaire
OCT: optical coherence tomography
RP: retinitis pigmentosa

## References

[1] Delyfer MN, Léveillard T, Mohand-Saïd S, Hicks D, Picaud S, Sahel JA (2004). Inherited retinal degenerations: therapeutic prospects. Biol Cell..

[2] Hartong DT, Berson EL, Dryja TP (2006). Retinitis pigmentosa. Lancet..

[3] Berson EL, Rosner B, Sandberg MA, Hayes KC, Nicholson BW, Weigel-DiFranco C (1993). A randomized trial of vitamin A and vitamin E supplementation for retinitis pigmentosa. Arch Ophthalmol..

[4] Berson EL, Rosner B, Sandberg MA, Weigel-DiFranco C, Moser A, Brockhurst RJ (2004). Clinical trial of docosahexaenoic acid in patients with retinitis pigmentosa receiving vitamin A treatment. Arch Ophthalmol..

[5] Li T, Sandberg MA, Pawlyk BS, Rosner B, Hayes KC, Dryja TP (1998). Effect of vitamin A supplementation on rhodopsin mutants threonine-17/methionine and proline-347/serine in transgenic mice and in cell cultures. Proc Natl Acad Sci U S A..

[6] Clemson CM, Tzekov R, Krebs M, Checchi JM, Bigelow C, Kaushal S (2011). Therapeutic potential of valproic acid for retinitis pigmentosa. Br J Ophthalmol..

[7] Siqueira RC (2011). Stem cell therapy for retinal diseases: update. Stem Cell Res Ther..

[8] Siqueira RC, Messias A, Voltarelli JC, Scott IU, Jorge R (2011). Intravitreal injection of autologous bone marrow-derived mononuclear cells for hereditary retinal dystrophy: a phase I trial. Retina..

[9] Siqueira RC. Autologous bone marrow-derived stem cells transplantation for retinitis pigmentosa (RETICELL). Clinicaltrial identifier: NCT01560715 [Internet]. Bethesda (MD): National Library of Medicine, 2012, Available from: https://clinicaltrials.gov/ct2/show/NCT01560715?term=siqueira&rank=4 (cited 15 April 2012).

[10] Mangione CM, Lee PP, Pitts J, Gutierrez P, Berry S, Hays RD (1998). Psychometric properties of the National Eye Institute Visual Function Questionnaire (NEI-VFQ). NEI-VFQ Field Test Investigators. Arch Ophthalmol..

[11] Mangione CM, Lee PP, Gutierrez PR, Spritzer K, Berry S, Hays RD (2001). Development of the 25-item National Eye Institute Visual Function Questionnaire. Arch Ophthalmol..

[12] Messias K, Jägle H, Saran R, Ruppert AD, Siqueira R, Jorge R (2013). Psychophysically determined full-field stimulus thresholds (FST) in retinitis pigmentosa: relationships with electroretinography and visual field outcomes. Doc Ophthalmol..

[13] Siqueira RC, Messias A, Voltarelli JC, Messias K, Arcieri RS, Jorge R. Fixation stability and central retinal sensitivity after intravitreal autologous bone-marrow stem cells for hereditary retinal dystrophy. In: ARVO, 2012, Fort Lauderdale. ARVO Meeting Abstracts, vol. 53. 2012. p. 6432.

[14] Arcieri RS, Messias K, Castro VM, Siqueira RC, Jorge R, Messias A. Intravitreal autologous bone-marrow stem cells in retinitis pigmentosa patients: one-year results. ARVO 2013. Invest Ophthalmol Vis Sci. 2013;54: E-Abstract 643.

[15] Seo JH, Yu HG, Lee BJ (2009). Assessment of functional vision score and vision-specific quality of life in individuals with retinitis pigmentosa. Korean J Ophthalmol..

[16] Sugawara T, Sato E, Baba T, Hagiwara A, Tawada A, Yamamoto S (2011). Relationship between vision-related quality of life and microperimetry-determined macular sensitivity in patients with retinitis pigmentosa. Jpn J Ophthalmol..

[17] Park SS, Caballero S, Bauer G, Shibata B, Roth A, Fitzgerald PG (2012). Long-term effects of intravitreal injection of GMP-grade bone-marrow-derived CD34+ cells in NOD-SCID mice with acute ischemia-reperfusion injury. Invest Ophthalmol Vis Sci..

